# Epidemiological outcomes and policy implementation in the Nordic countries during the COVID-19 pandemic

**DOI:** 10.1186/s13690-025-01531-5

**Published:** 2025-02-20

**Authors:** Adam Hallberg, Mia Aakjaer, Katri Aaltonen, Morten Andersen, Elisabeth Pedersen, Mohammadhossein Hajiebrahimi, Hedvig Nordeng, Fredrik Nyberg, Per-Jostein Samuelsen, Björn Wettermark

**Affiliations:** 1 Department of Pharmacy, Uppsala University, Box 580, Uppsala, 751 23 Sweden; 2 Pharmacovigilance Research Center, Department of Drug Design and Pharmacology, University of Copenhagen, Copenhagen, Denmark; 3 INVEST Research Flagship Center, University of Turku, Turku, Finland; 4 Pharmacoepidemiology and Drug Safety Research Group, Department of Pharmacy, Faculty of Mathematics and Natural Sciences, University of Oslo, Oslo, Norway; 5 School of Public Health and Community Medicine, Institute of Medicine, Sahlgrenska Academy, University of Gothenburg, Gothenburg, Sweden; 6 Regional Pharmacovigilance and Medicines Information Centre (RELIS), University Hospital of North Norway, Tromsø, Norway; 7 Pharmacy and Pharmacology Centre, Faculty of Medicine, Vilnius University, Vilnius, Lithuania

**Keywords:** COVID-19, Policy, Mortality, Hospitalization, Nordic countries

## Abstract

**Background:**

During the initial phase of the COVID-19 pandemic, there was an intensive debate on which strategies would be most effective to minimize the negative societal impact of the pandemic. This study aimed to provide an overview of key epidemiological outcome measures of the disease in the Nordic countries and the subsequent policy implementation that were undertaken to curb the outbreak.

**Methods:**

Time trends in test-positive infections, hospitalizations, and intensive care unit (ICU) admissions due to COVID-19 as well as COVID-19 mortality and excess mortality were compared between Denmark, Finland, Iceland, Norway, and Sweden. The epidemiological patterns were presented in relation to 13 different policies implemented to a different degree in the countries, eight of which were related to containment and five to health systems policy. A stringency index summarized the intensity of the policies. Data were collected from Our World in Data, the Oxford COVID-19 Government Response Tracker and Eurostat. The investigated time period was 1 January 2020 to 30 April 2022.

**Results:**

Overall, Sweden had more infections, deaths, hospitalizations, and ICU admissions than the other Nordic countries during the first three waves of the pandemic. However, in the fourth wave, Denmark exceeded Sweden in all outcomes. The overall stringency among the Nordic countries varied broadly. The lowest average stringency index was observed in Iceland and the highest in Sweden. Excess mortality over the whole study period was lowest in Iceland while Norway had very few ICU admissions.

**Conclusions:**

The Nordic countries took vastly different approaches to contain the spread of the pandemic, but the long-term impact on excess mortality was similar. The variety in policy responses and epidemiological measures bring many opportunities for learning across the countries.

**Supplementary Information:**

The online version contains supplementary material available at 10.1186/s13690-025-01531-5.

**Table:** 

Text box 1. Contributions to the literature
• The Nordic countries share many similarities in terms of population demography and health systems, but they took very different approaches during the Covid-19 pandemic.
• There are limited scientific papers available visualizing the interplay between policy measures and epidemiological outcomes during the Covid-19 pandemic.
• Even though the complexity and rapid changes in policies does not allow for any evaluation of the impact of single policies, visualization may provide opportunities for learning across the five countries to plan for future policy decisions.

## Background

The first Corona virus disease 2019 (COVID-19) infections were detected in Wuhan, China, in December 2019 and the epidemic quickly spread around the world. In January, when the news about the novel coronavirus spread, the Nordic countries took various actions. Iceland directly enacted its preparedness act and in mid-February physical distancing and travel restrictions were implemented in Norway [1, 2]. Yet, the first case in the Nordic countries was detected in Finland on 29 January 2020[3]. Sweden detected the first case the next day [4]. Norway and Denmark reported their first cases one month later, on 26 February, and the World Health Organization (WHO) declared COVID-19 a pandemic on 11 March 2020 [2, 5]. On 28 February 2020 Iceland reported its first infection [1]. Denmark and Norway began imposing restrictions to limit the spread of COVID-19 and implemented lockdown measures on 12 March 2020, two weeks after detecting their first cases. In mid-March, Finland used its Emergency Powers act and Denmark used their National Preparedness act to enact restrictions, such as bans on international travel and reduced social interactions. Finland also had internal movement restrictions in place for approximately three weeks from 27 March 2020 to 15 April 2020 [6]. Sweden, on the other hand, initially relied mainly on recommendations to the general public [4]. As in the other Nordic countries, communication mainly took place through press briefings. There was no formal lockdown in Sweden, but strict hand hygiene and physical distancing was early recommended by authorities [4]. On 13 March 2020, as the first Nordic country, Iceland began screening individuals, and the initial study showed that COVID-19 to a large extent was asymptomatic [1].

Globally, the pandemic led to extraordinary policy responses, that have been broadly categorized into initial conversion towards cautionary actions through closure and containment policies, subsequent differentiating stringency across countries related to stay-at-home policies, and further increasing heterogeneity in approaches across countries and subnational regions [7]. In retrospect, restrictive policy measures have been associated with reduced transmission rates and fatalities, but they have also been blamed for negative consequences on mental health, and economic disruptions; however, with a large heterogeneity in impact across studies [8–14].

The differences between countries must be considered when judging policies implemented to mitigate the pandemic regarding the outcome in terms of e.g. number of cases and deaths observed and the timing of such outcomes. The initial lack of large-scale coordinated containment/suppression measures such as school closings, limitation on gatherings, and physical distancing in Sweden made it an outlier compared to most other European countries in both pandemic response and initial impact on population health [6, 15]. While some previous articles have addressed the question of Sweden as a policy outlier, they focused on the first wave or early pandemic [5, 6, 15–18]. Other researchers have focused on the economic trade-offs of certain policy decisions [19] and health system resilience, comparing up to 30 countries [20]. There are also other policy evaluations comparing different policy perspectives between one or two Nordic countries and others [21–25]. Other research has compared excess mortality across countries but has not included any comparative data on policy measures [26]. To the best of our knowledge, there is no comprehensive overview of both epidemiological outcome measures and policies implemented in all the five Nordic countries. Such an analysis would provide a valuable platform for learning across countries, to plan for future policy decisions or more in-depth evaluations of the effectiveness of different strategies.

The aim of this study was to describe the evolution of the COVID-19 pandemic in Denmark, Finland, Iceland, Norway, and Sweden by comparing major policy implementations in each country to mitigate the spread of the disease, with key epidemiological outcome measures from each country on test-positive infections, hospitalizations, and ICU admissions due to COVID-19, COVID-19 mortality, and excess mortality.

## Methods

### Study design and setting

This was an ecological study comparing time trends in epidemiological outcomes of the disease (infection rates, hospitalizations, ICU admissions, and deaths due to COVID-19) with policies implemented to manage COVID-19 in Denmark, Finland, Iceland, Norway, and Sweden. The study included data from all five Nordic countries, which together have a population of twenty-seven million people. A summary of key data regarding population demography, healthcare spending, and availability of healthcare professionals in the countries is presented in Supplementary Table S1.

The Nordic countries share many similarities in terms of population, demographic characteristics, and how their health systems are structured. All countries have a tax-funded health care system with universal coverage for all residents [27]. Health care is decentralized and regulated at different levels, with the national level setting strategic policies, legislation and framework for the healthcare system [24]. However, the extent to which the governance has been decentralized to regions and municipalities varies substantially between the countries [6]. Additional information about the health systems in the Nordic countries can be found as Supplementary material and in the paper by Laugesen et al. 2021 [27].

### Data sources and outcome measures

Data was collected from Our World in Data (OWID) and the Oxford COVID-19 Government Response Tracker (OxCGRT). Both data sources are available under a CC BY 4.0 license [28]. OWID compile data collected from national public health agencies, WHO COVID-19 data, the European Centre for Disease Prevention and Control (ECDC), the World Bank, the Organisation for Economic Co-operation and Development, and JHU Center for Systems Science and Engineering (CSSE) among others on nine different markers. The full technical documentation has been explained elsewhere [29].

The OxCGRT collected its data from publicly available data such as news articles, government press releases, and briefings [30].

We assessed four epidemiological outcomes: Infections, hospitalization, ICU admissions, and deaths due to COVID-19. OWID, in turn, collected data on infections and deaths from the WHO. We defined a confirmed infection of COVID-19 in this study as a person confirmed with any laboratory test that is in accordance with WHO guidelines, regardless of symptoms [31]. Data for ICU and hospitalization according to OWID were collected from the local authorities in three countries: Statens Serum Institut in Denmark, National Board of Health and Welfare in Sweden, and The Finnish Institute for Health and Welfare, respectively. For Iceland and Norway, OWID collected their data from the European Centre for Disease Prevention and Control.

The OxCGRT data for policy was scored by OxCGRT and the degree of intensity in each type of policy were rated as “policy level” rated from zero to a maximum level of 2 to 5 depending on the type of policy (see further below). Their method and basis for the scoring has been described elsewehere [32]. Researchers at Oxford University at the Blavatnik School of Government manage the OxCGRT database. The definitions of the OxCGRT were harmonized for all countries but may be different from the formal definitions were used in national statistics in each country, for example, testing policy data for Finland does not agree with the Finnish definitions. Excess mortality data was collected from Eurostat and a detailed technical outline has been described [33]. The Eurostat data were collected weekly from April 2020 with the specific aim to “support the policy and research efforts related to COVID-19.” [30] Excess death was defined as “the rate of additional deaths in a month compared to the average number of deaths in the same month over a baseline period”. The baseline period set by Eurostat was defined as 2016–2019 not adjusted for age but for coverage (incompleteness) [34] The granularity of the excess mortality data for Eurostat is monthly while the data provided by OWID and OxCGRT are daily data points.

We define a COVID-19 wave as an upward and/or downward period in infection occurrence that is substantially sustained over a period of time, which distinguishes a wave from an uptick, a downtick, reporting errors, or volatility in new cases [35]. The common dates selected to delimit COVID-19 waves for this study across the five countries are presented in Supplementary Table S2.

### Investigated policies

Eight different containment policies and five health system policies were investigated in this study (Supplementary Table S3). Policies were scored from 0 to increasing stringency by integers. For example, school closings were scored 0–3 (where 3 indicated requiring closing at all levels), while restrictions on gatherings were scored 0–4 (where 4 indicated restrictions on gatherings of ≤ 10 people). For details, see Supplementary Table S3.

The “government response index” is an average over all indicators in the OxCGRT database and shows how the response has varied, becoming more or less strict in limiting people over the study period. We used the government response index to get an overall composite view even though it also contains economic policies as factors. For details on how the index is calculated, see the original reference [29].

### Statistical analyses and visualization

For all policies investigated, a timeline was constructed using the OxCGRT data. Graphs were generated using Microsoft Excel and the countries were separated using a ± 0.05 spacing difference, meaning that 3.05 = 2.95 = 3. For the epidemiological outcomes, we created timelines using data from OWID and since the fourth wave had more infections than previous waves, the infection timeline was separated into two panels.

### Ethical considerations

The data was entirely based on published and publicly available aggregate data. Consequently, no ethical approval was needed for this study.

## Results

**Fig. 1:**
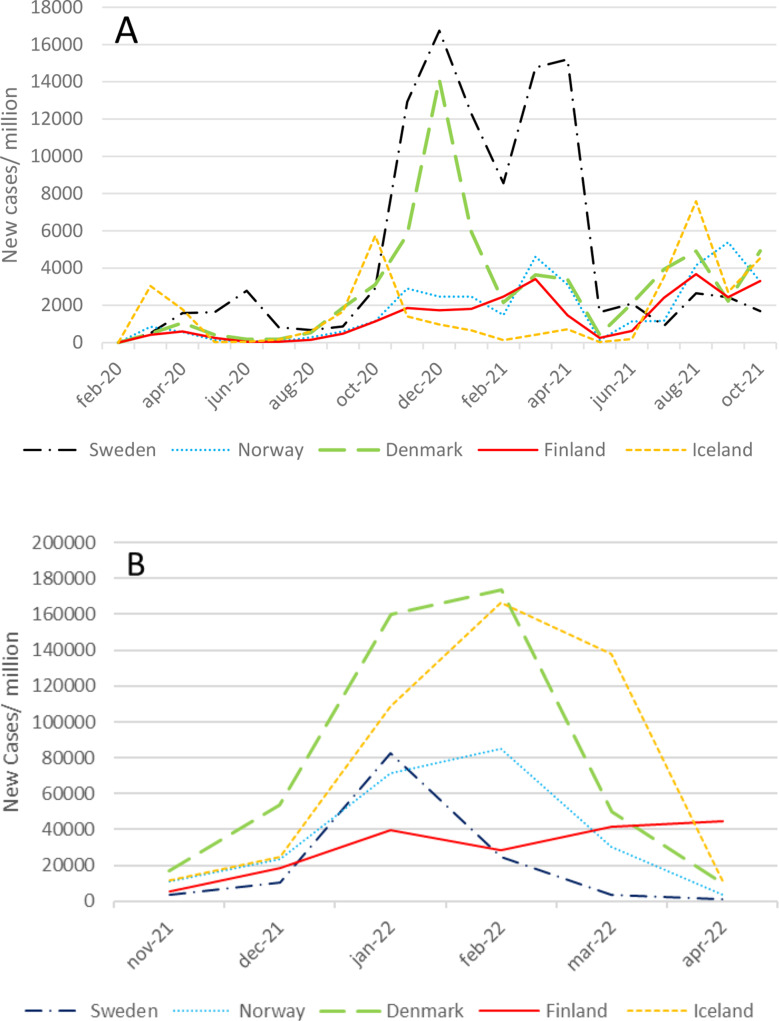
Number of Corona virus disease 2019 positive infections per month in the first three waves (**A**) and during the fourth wave (**B**)

The number of COVID-19 positive infections reported during the four waves are visualized in Fig. 1. Sweden reported more infections per million inhabitants than any other Nordic country in the first three waves. In the fourth wave, Denmark and Iceland reported more infections per million inhabitants than the other Nordic countries. The Nordic countries experienced waves of infections, hospitalizations, deaths, and ICU admissions at slightly different times depending on when and how the virus arrived and spread within the country.

**Fig. 2:**
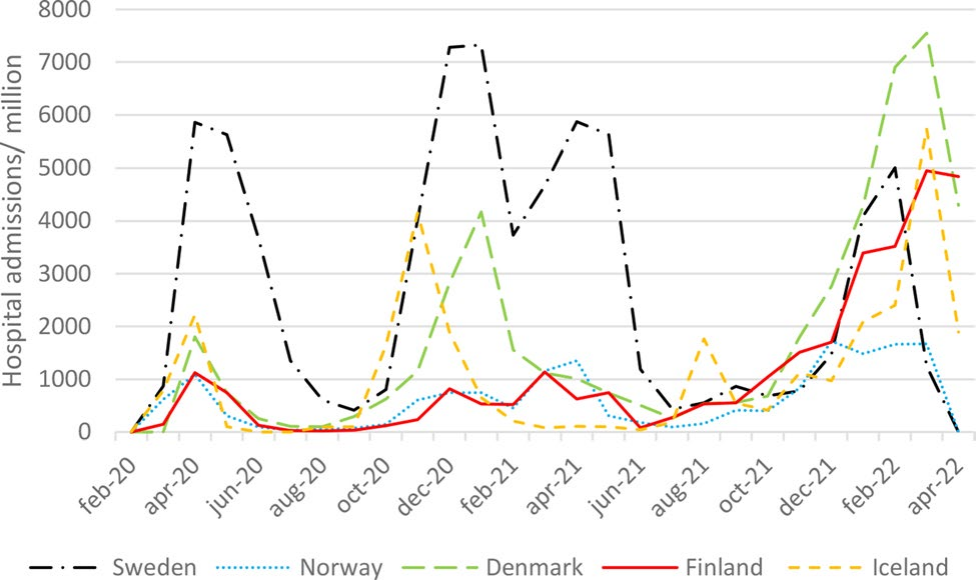
Hospital admissions per million inhabitants and month during the COVID-19 pandemic

While there were more inpatient hospitalizations per million inhabitants in Sweden during the first three waves, Denmark became the top country in the fourth wave (Fig. 2). Both Finland and Norway had low levels of inpatient hospital admissions during the initial phases of the pandemic, but there was a rise in hospitalizations in Finland during the fourth wave. Iceland had peaks mainly in the second and fourth waves.

**Fig. 3:**
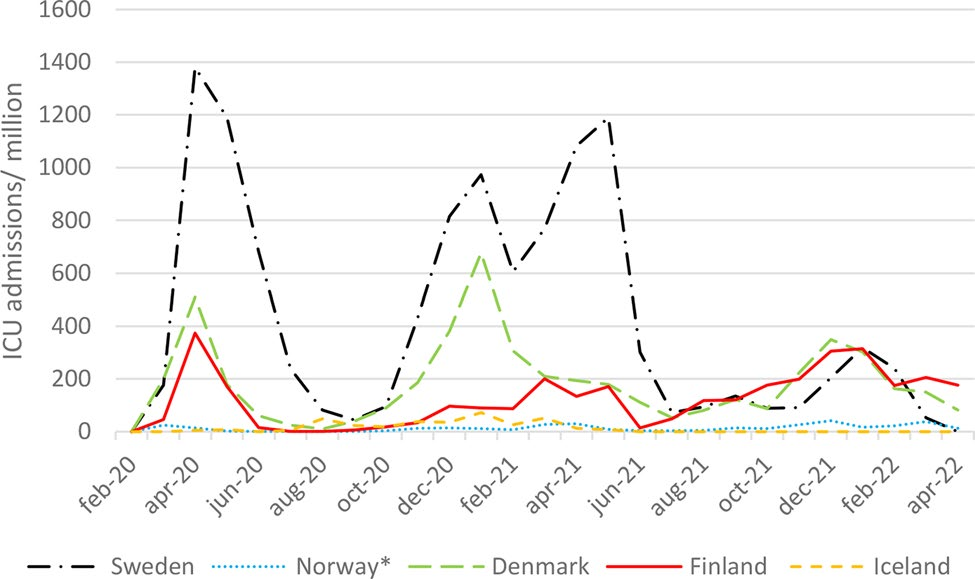
Intensive care unit (ICU) admissions per million inhabitants and month during the COVID-19 pandemic. * Norwegian data was obtained from the Norwegian Institute of Public Health

Compared to Denmark, Finland and Sweden; Norway and Iceland did not have as many ICU admissions. The greatest increase in ICU admissions was observed in Sweden followed by Denmark (Fig. 3).

**Fig. 4:**
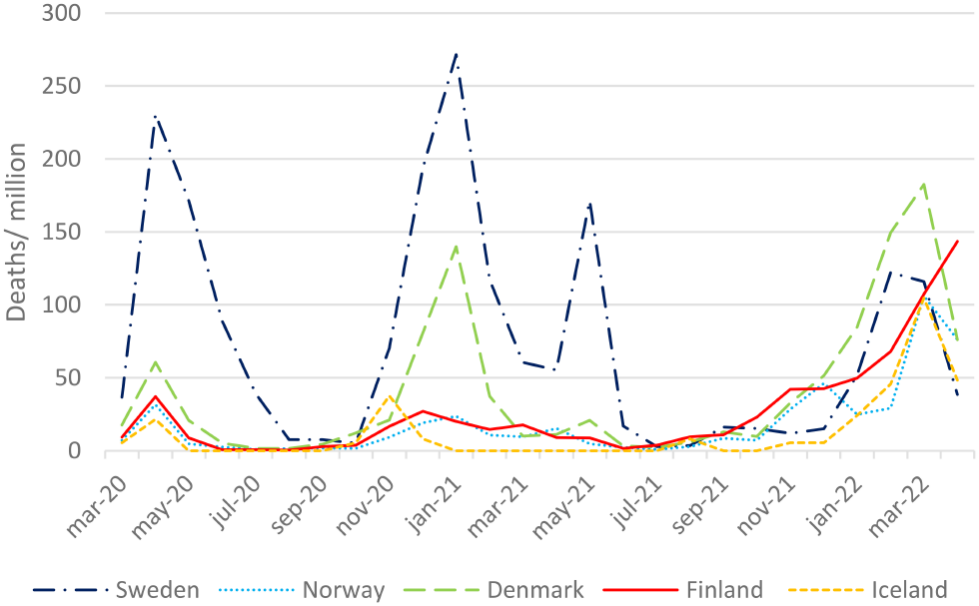
Deaths per month with deaths attributed to COVID-19, per million inhabitants, during the COVID-19 pandemic

Sweden had a higher number of deaths per million inhabitants compared to the other Nordic countries during the first three waves. During the fourth wave, Denmark had the highest number of deaths per million (Fig. 4). In Finland, mortality increased rapidly towards the end of the observation period.

**Fig. 5:**
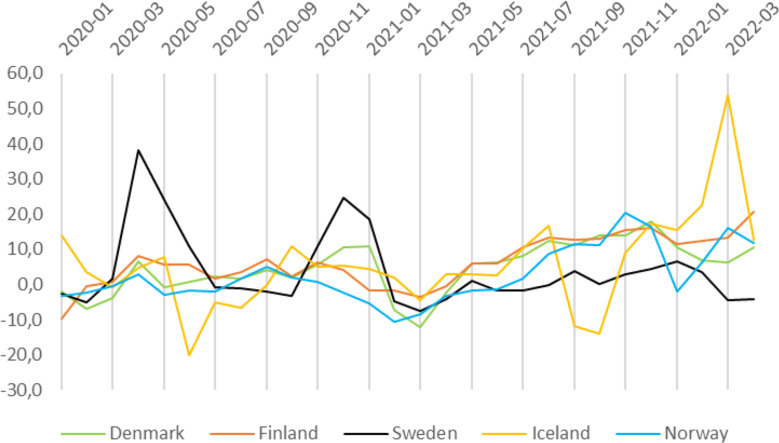
Monthly excess mortality in the Nordic countries during the COVID-19 pandemic. Percentage of additional deaths in each month compared to 2016–2019 average mortality for the same month

Excess mortality relative to 2016–2019 average mortality as reported by Eurostat also varied substantially over time and between the Nordic countries (Fig. 5). Sweden had the highest excess mortality at the beginning of the pandemic, while Iceland had the highest excess mortality towards the end of the study period. The different waves of the pandemic are discernible in the excess mortality data. We observed a negative excess mortality in all Nordic countries at some point during the study period.

### Health system policy measures

**Fig. 6:**
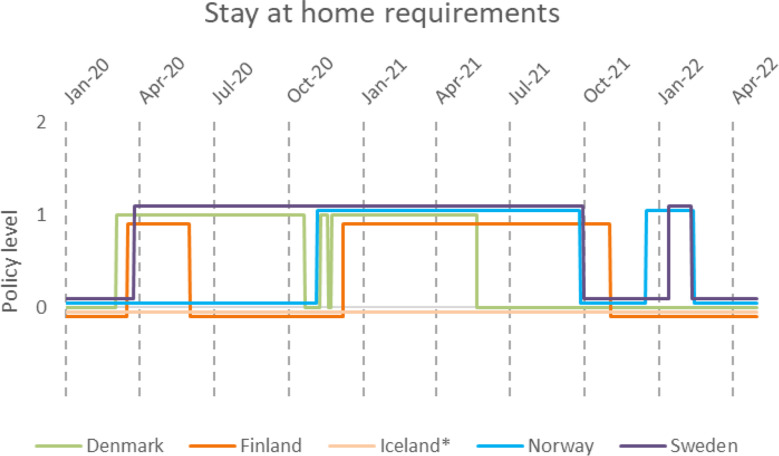
Stay-at-home or shelter-in-place policy. 0- No measure, 1-Recommend not leaving home. 2-Require not leaving home with exceptions for daily exercise, grocery shopping and essential trips. 3- Require not leaving the house with minimal exceptions

All Nordic countries except for Iceland recommended people to stay at home but for different periods (Fig. 6). No country went beyond recommending staying at home and, consequently, no Nordic country applied enforcement for people to stay at home linked to any legal measure.

**Fig. 7:**
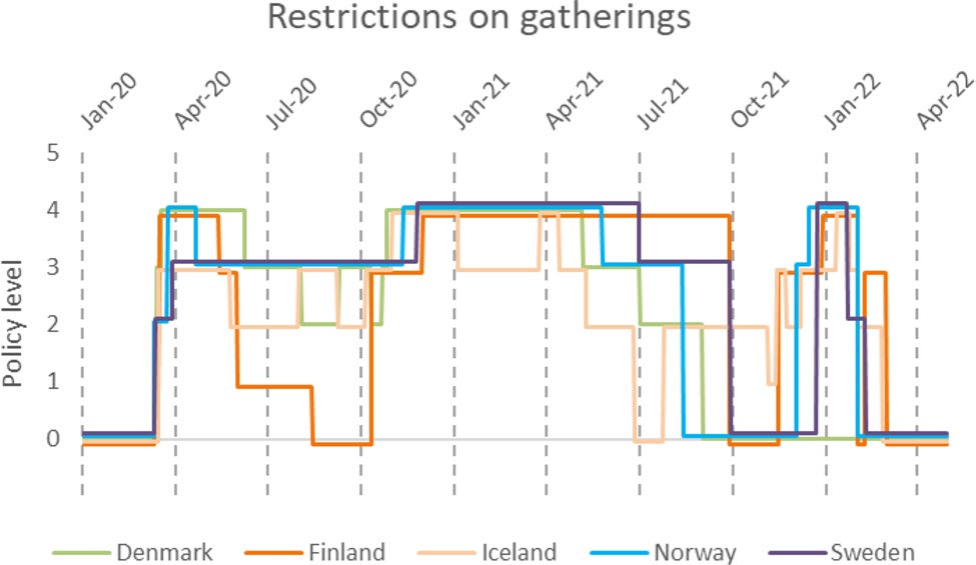
Changes in restriction on gatherings policy. 0-No measure, 1-Restrictions on very large gatherings (> 1000 people) 2-Restrictions on gatherings between 101–1000 people, 3-Restrictions on gatherings between 11–100 people, 4-Restrictions on gatherings of ≤ 10

The restrictions on gatherings also varied between countries (Fig. 7). Yet, at some point in time all countries imposed the most restrictive policy, limiting gatherings of more than ten people.

**Fig. 8:**
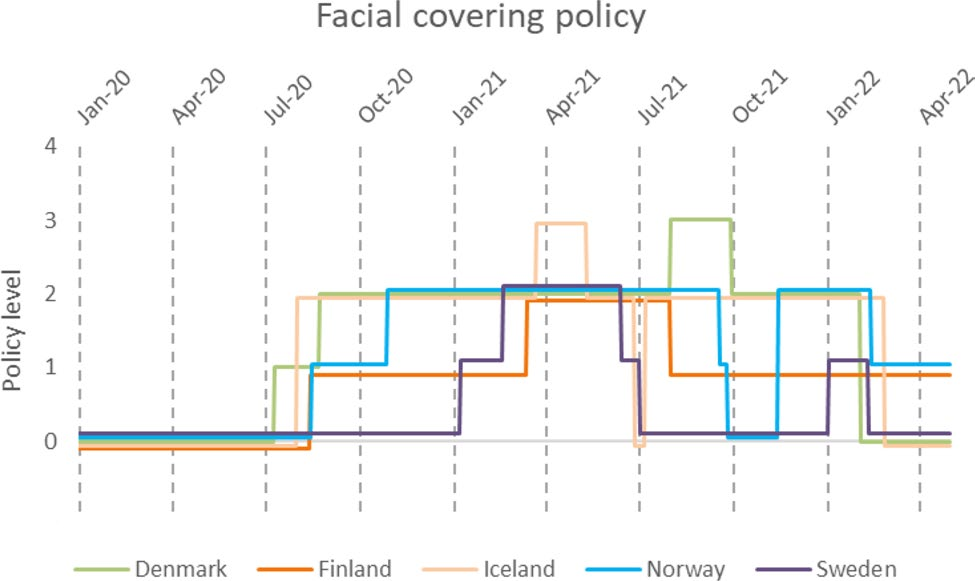
Change in masking policy in the Nordic countries during the COVID-19 pandemic, according to time and policy level. 0-No policy, 1-Recommended, 2-Required in some specified public places when physical distancing is not possible. 3-Required in all public places when physical distancing is not possible. 4-Required outside the home at all times regardless of location or presence of people

No Nordic country ever implemented a masking policy at level four, requiring masks to be worn outside the home regardless of location or presence of people (Fig. 8). At the end of the fourth wave, only Finland and Norway still recommended masking. The first country to enact a policy on masking was Denmark, closely followed by the other Nordic countries, except Sweden, which recommended masking in January 2021.

**Fig. 9:**
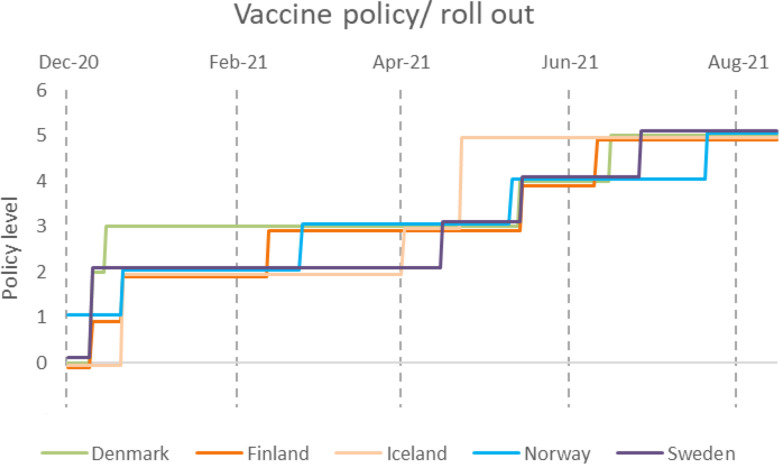
Vaccination rollout in the Nordic countries according to time and policy level. 18 December 2020 to 1 September 2021, from there on out the vaccine is available at policy level 5 in all Nordic countries. 0-No vaccine available, 1-Available to one of the following groups: key workers / clinically vulnerable groups / elderly, 2-Available to two of the aforementioned groups, 3-Available to all aforementioned groups, 4-Available to all groups plus partially available to select broad groups and ages, 5-Universal availability

Vaccination access was similar between the Nordic countries and by 7 August 2021, it was extended to all persons above 18 years of age (Fig. 9). Norway was the first Nordic country to make the vaccines available. On 27 December 2020, the first dose was administered in both Sweden and Norway. Denmark was more rapid to make the vaccine available for all essential workers, clinically vulnerable, and elderly persons. Other Nordic countries more gradually reached their targets for vaccination coverage. All Nordic countries provided the vaccine at no cost to the individual.

**Fig. 10:**
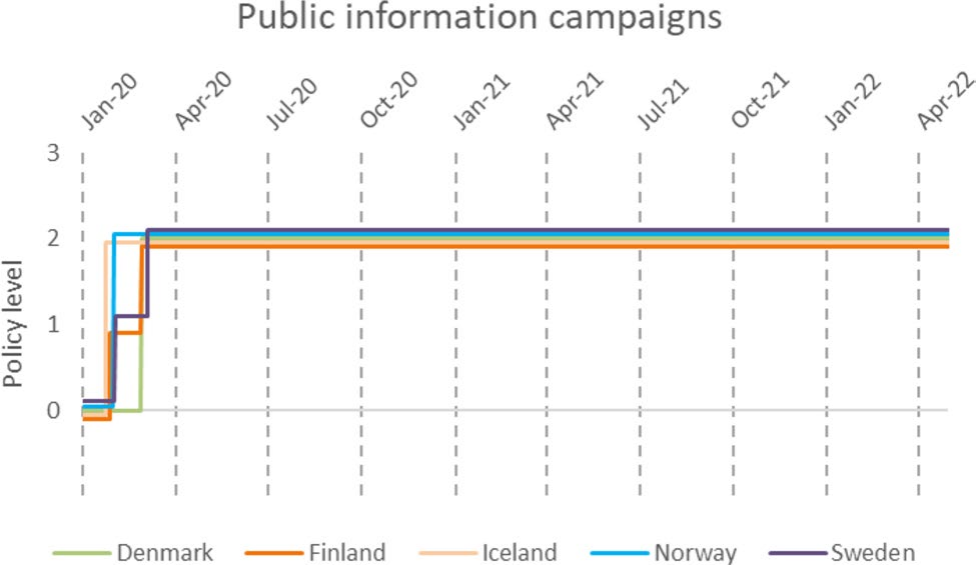
Public information campaigns in the Nordic countries during the COVID-19 pandemic, according to time and policy level. 0-No campaign, 1-Public officials urging caution, 2-Coordinated public information campaigns

**Fig. 11:**
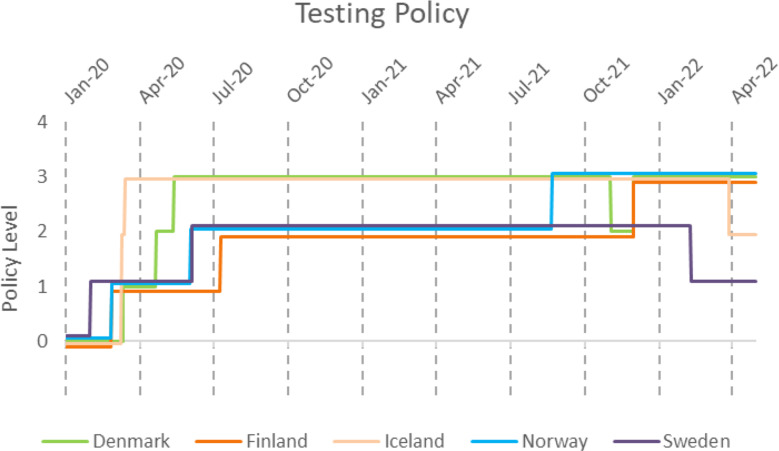
Implementation of testing policy in the Nordic countries during the COVID-19 pandemic, according to time and policy level. 0-No testing policy, 1-Only those who have symptoms and meet specific criteria such as key workers, inpatients, part of contact tracing. 2-Testing of anyone with symptoms, 3-Open public testing

**Fig. 12:**
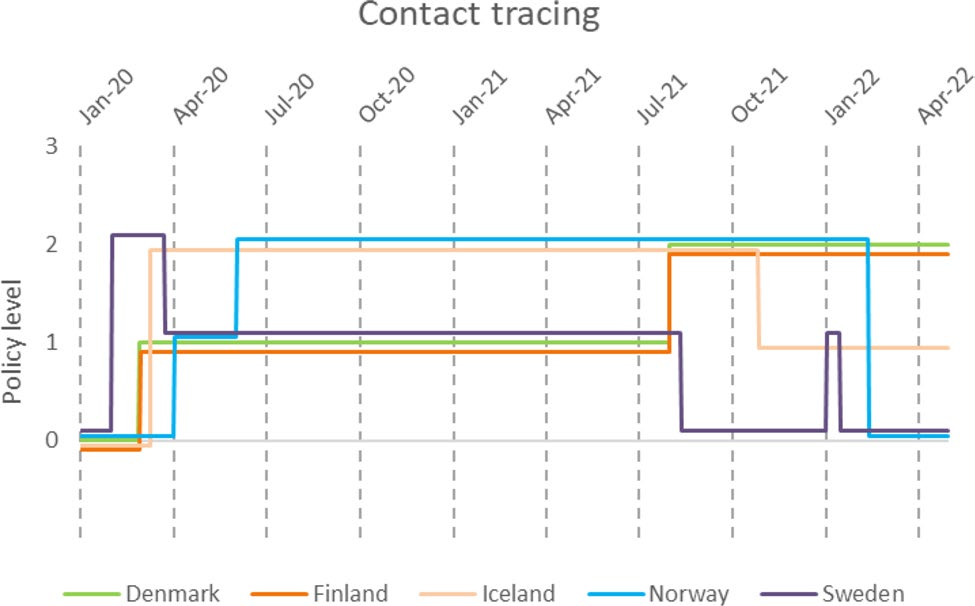
Implementation of contact tracing policy in the Nordic countries during the COVID-19 pandemic, according to time and policy level. 0- No contact tracing, 1-Limited contact tracing not done for all infections. 2-Comprehensive contact tracing done for all identified infections

Public information campaigns (Fig. 10) were quickly introduced along with testing strategies (Fig. 11) and contact tracing (Fig. 12) in all countries. However, there were quite a lot of differences in testing and contact tracing policy implementations between countries and even within countries, e.g. Swedish regions.

Iceland and Denmark were first to impose a level three testing policy, which meant that testing was available to anyone with or without symptoms (Fig. 11). Sweden did not have a testing policy where anyone who wanted could be tested; rather, only symptomatic people and individuals identified via contact tracing or in risk occupations such as health care were recommended to get tested. However, in practice, if you went to a testing point and claimed to have symptoms you were tested. According to the OWID data, Finland implemented universal testing during the fourth wave. However, based on qualitative description on policy responses, at least some restrictions (e.g., to symptomatic, exposed, specific occupations) remained throughout the observation period [28]. Sweden ended contact tracing after the third wave and Norway after their peak during the fourth wave (Fig. 12). Denmark, Finland, and Iceland all kept some level of contact tracing throughout all four waves. Finland reduced testing and tracing capacity to ease the burden on the healthcare system since September 2021.

### Containment and restrictive policy

**Fig. 13:**
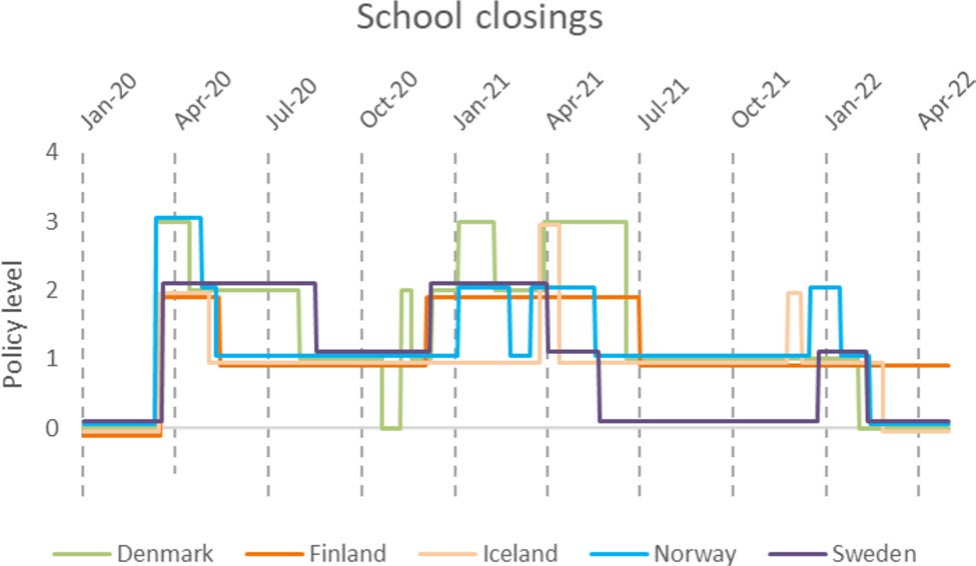
Changes in school closing policy. 0-No measure, 1-Recommended closing, 2-Required closing at some level, 3-Required closing all levels

We investigated eight policies related to disease containment and limiting people-to-people transmission. All eight policies were used to varying extents and varying strictness in the Nordic countries. Two policies that have received much attention in the public debate were school closings and restrictions on gatherings (Figs. 7 and 13). In the case of restrictions on gatherings, we observed fluctuating policy implementation with great variability both within and between countries (Fig. 7). In the case of school closings, observations indicated a similar non-consensus, with large variability in response, albeit less so than for restrictions on gatherings (Fig. 13). An interesting comparison between school and workplace closings can be observed with similar restriction levels during the first wave of March through April 2020 (Fig. 13 and S1) followed by a second restricting of both policies corresponding to the third wave. The closing of public transportation fluctuated much less and reached an overall lower score compared to restrictions on gatherings. Iceland for instance had a very brief period with public transport restrictions, only reaching a level 1 for less than 90 days while Sweden kept level 1 for more than 450 days (Figure S5).

Supplementary Figures S6-S15 show epidemiological outcomes together with a stringency index which ranges from 0 to 100. Norway (75.9), Denmark (72.2), and Finland (67.3) all ranked higher than Sweden (32.4) in terms of stringency index in March 2020.

## Discussion

In this study, we found that the Nordic countries used a large range of approaches to mitigate the COVID-19 pandemic with subsequent differences in epidemiological outcomes. All Nordic countries imposed physical distancing, limited the number of people at public gatherings and restricted international travel. However, the level, timing and duration of each policy varied heavily in each Nordic country. Our study showed that Sweden may have lost some time and control initially, partly due to a different legal framework and healthcare/care structure, but had equal or even better results on disease-spreading and excess mortality in the long term.

Our study confirms previous findings [17] that Sweden initially had more COVID-19 infections and deaths compared to other Nordic countries, which may be related to less strict policies initially during the pandemic. Sweden’s approach was questioned by some observers during the early pandemic for relying too much on recommendations instead of strict policy rules, but our study shows that the country does not seem to have fared appreciably worse in the long run with respect to excess mortality. However, different assessments of excess mortality come to somewhat different conclusions in this regard [37, 38]. Sweden and Iceland both had quite high excess mortality during certain time periods, Sweden had up to 40% in March 2020 and Iceland had over 50% in March 2022. For Iceland, this can be explained by the small population size, resulting in larger random variation. The large number of hospitalizations and high mortality in Denmark during the fourth wave (late 2021 to early 2022) reflects that a very large part of the adult population (59%) were infected with the omicron variant [39]. Omicron was not associated with more severe disease, but in the mortality surveillance numbers are “death with COVID-19”, i.e., counting all deaths during 30 days after COVID-19 (ref) [40]. It shows that the evaluation based on death certificates (deaths caused by COVID-19) gives much lower numbers, emphasizing the importance of exact definitions when you analyze data over time and between countries. In Finland, mortality increased rapidly since the end of 2021, and in 2022, it contributed to decreasing life expectancy. This seems partly attributed to the COVID-19; however, other reasons such as changes in the population age structure may have also played a role [41]. A longer comparison of Denmark, Finland, Norway, and Sweden showed relatively low excess mortality in all countries, but substantial variation in the timing [42]. Overall, analyses of excess mortality have many methodological challenges and assessments of, e.g., seasonal variations [43] and regional differences within a country [44] have added some insights. It will also depend on the selected baseline, and it has been suggested that in 2020, Sweden’s excess mortality reversed health gains to levels seen during 2017-2018 [45].

We also found large variations between the Nordic countries in policy and decision-making. There are many reasons for the different approaches including differences in sociodemography, legislation and healthcare systems. Early during the pandemic, it became clear that controlling contact rates was a key to outbreak control, and strategies should be adapted to population densities [45]. Later studies have confirmed that population density and human mobility had a significant impact on infections rates and deaths, but there has so far been more significant associations between geography and COVID-19 within than between countries [46]. Overall, the Nordic countries have sparse population densities compared to other countries, with Iceland having the added benefit that it is geographically isolated as an island.

Some differences between the Nordic countries in policies implemented should also be viewed in relation to contextual factors around the health system and the role of different stakeholders. The Swedish legal framework did at the time not allow a “state of emergency”, except for in case of war [47, 48]. Other Nordic countries on the other hand had this option and both Finland and Norway declared it early on. So even though similar responses were enacted the extent to which the different governments could enforce them varied significantly. Earlier studies have shown that Finland and Denmark had strong political influence in their decision-making, while Norway, Iceland, and Sweden relied more heavily on expert advice that was freer from political interference thus leading to more network-based governance [22].

It would have been valuable to assess which of all the different policies implemented in the Nordic countries at different time points that had an impact on the disease spreading. This is, however, not feasible to do given the multiple policies implemented concomitantly and the rapid changes in the nature of them, partly done as responses of a rapid spreading of the infection and partly in anticipation of preventing such spread. However, laying out the policies and outcomes side by side as done here provides some basis for an informed discussion. There is also some evidence from the literature on the effect of some policies. A review of 18 studies from different countries in Asia, Europe and North America, conducted early during the pandemic, suggested that travel restrictions, quarantine of travelers, city lockdown, restrictions of mass gathering, physical distancing measures, compulsory mask wearing, contact tracing and testing, school closures and personal protection among health workers were effective in mitigating the spread of COVID-19 [49]. The quality of some studies were, however, rated as rather low, partly due to the problems raised above.

Finally, vaccination uptake is important to quell a pandemic and a recent study showed that European countries rapidly providing vaccination and booster administration had lower excess mortality that European countries that were slower to immunize their citizens [50]. Sweden achieved a slightly lower vaccination uptake rate (73%) by beginning of 2022 than the other Nordic countries (81‒84%) [51]. Another study showed interesting differences between the Nordic countries in vaccine coverage over time and in special population groups [52]. However, compared to other countries, all the Nordic countries performed rather well in terms of vaccinations [53], which may be attributable to a history of trust toward institutions and a high education level enabling informed decisions. Further analyses of the impact of country variation in vaccination rates in different population strata on the epidemiological outcomes would be interesting, but is not feasible within this ecological study design using only aggregated data. Such studies would require more analytical epidemiological methods using individual level healthcare data on vaccinations, risk factors and outcomes, which might be feasible using the rich Nordic population-based registers [27, 54].

### Strength and limitations

We believe our study adds insights to previously published studies since it particularly highlights the interplay between policy and subsequent epidemiological outcomes. While we have not done a specific evaluation of the effect of certain policies, we have provided a comprehensive overview of epidemiological measures and the large range of different policies over essentially the entire pandemic period. We used open-access aggregated standardized datasets collected from government agencies about the spread of the virus and the evolution of the public health measures [55]. A similar methodology (e.g., stringency index, policy levels) enabled comparisons.

However, we also acknowledge some limitations. Iceland and Norway have fewer people aged over 70 years (9.9% and 12.6%, respectively) compared to the other Nordic countries [18]. This has not been accounted for in the analysis of excess mortality used here. Furthermore, the observed difference in excess mortality might vary depending on which baseline periods that are used, something that is also mentioned by Kepp et al. [37]. Apart from vaccines, the policies we investigated did not include specific information on interventions aiming at protecting high-risk groups, that may have influenced the outcomes and mortality in vulnerable persons such as the older population.

There was quite limited testing availability at the start of the pandemic, something which may have contributed to an under-reporting of infections [56–58]. Data on reported infections during the final wave are also not comparable to previous waves, because of milder symptoms and increasing use of home testing, and changes in the testing and contact tracing strategy in the beginning of 2022.

While the epidemiological outcome measures to large extent are standardized and have been validated and used in large numbers of scientific papers, the policy ratings are more subjective. There may also be large variations within the Nordic countries not captured in national assessments as implemented by OxCGRT. Many policies (school closures and internal movement restrictions for example) were implemented differently across regions, depending on the number of infections. Thus, a better understanding of the covariation between policies and epidemiological outcomes would require more detailed within-country regional comparisons.

The reliability of the OxCGRT policy data is particularly questionable for Finland, where the dates and levels do not always seem in line with other evidence. This may be due to limitations of the data collection method in capturing within-country heterogeneity. In the Finnish case, the decentralized model of management and distribution of tasks and responsibilities across different agencies and bodies is likely to have complicated summarizing evidence [3, 59]. Nevertheless, we used data as published by the OxCGRT, because changes would have undermined the main strength of the data: having been systematically collected from all countries they cover using the same methodology. Finally, although our study covered a long period, it is important to acknowledge that assessing the spreading of the infection and subsequent changes in mortality related is only one impact of a pandemic, and the total societal impact in terms of, e.g., increased burden of non communicable diseases, mental health problems, economical recession and increasing inequities remains to be systematically evaluated [60].

## Conclusion

This paper provides an overview of the COVID-19 pandemic and measures taken to mitigate the spreading of the infection in the five Nordic countries. Policymakers took vastly different approaches to contain the spread of the pandemic but the long-term epidemiological impact seems similar. The complexity and rapid changes in different policies does not allow for firm conclusions of the impact of single policies on the epidemiological outcomes, but the overview provide opportunities for learning across the five countries, to plan for future policy decisions or more in-depth evaluations of the effectiveness of different strategies.

## Electronic supplementary material

Below is the link to the electronic supplementary material.

Supplementary Material 1

## Data Availability

No datasets were generated or analysed during the current study.

## Abbreviations

COVID-19: Coronavirus disease 2019
GP: General practitioner
OWID: Our world in data
OxCGRT: Oxford COVID-19 government response tracker
ICU: Intensive care unit
WHO: World health organization
