# Supplementary material for: Epidemiological outcomes and policy implementation in the Nordic countries during the COVID-19 pandemic
Source: Arch Public Health. 2025 Feb 20;83:46. doi: 10.1186/s13690-025-01531-5 (PMC11844186; doi:10.1186/s13690-025-01531-5)
Supplement: Supplementary file 1 — Supplementary Material 1 [file 13690_2025_1531_MOESM1_ESM.docx]

**Supporting information**

| Supplementary Table S1: Key population and health characteristics of the Nordic countries; 2019 data^31^ | | | | | |
| --- | --- | --- | --- | --- | --- |
|  | Denmark | Finland | Iceland | Norway | Sweden |
| Population (million) | 5.8 | 5.5 | 0.4 | 5.3 | 10.3 |
|  |  |  |  |  |  |
| Life expectancy (years) | 81 | 82 | 83 | 83 | 83 |
| Persons aged >65 years (%) | 20 | 23 | 15 | 18 | 20 |
| Healthcare spending (% GDP) | 10.2 | 9.2 | 8.6 | 10.4 | 10.8 |
| Healthcare spending per capita ($ USD) | 6384 | 4605 | 5096 | 7065 | 6262 |
| Number of doctors per 1000 inhabitants | 4.4 | 3.6 | 4.5 | 5.2 | 4.3 |
| Total hospital beds per 1000 inhabitants | 2.6 | 3.4 | 2.8 | 3.5 | 2.1 |

*Supplementary information on the Nordic health care system*

Most services, such as hospital visits, visits to primary care, medications are predominantly financed through taxes; between three quarters and 85%. This is supplemented with additional co-payment of 15-20% for visits to general practitioners (GPs), outpatient care and hospital stays. Universal care and reimbursement schemes for medicines are generally provided with exceptions for dental care. The health care systems are regulated on three levels: national, dealing with economic framework, monitoring and legislation; regional, and municipal. The municipalities’ responsibilities vary between countries, but they have in general some responsibility for primary care, disease prevention and nursing homes, except from Iceland, which does not have municipalities. In Finland, the municipalities were responsible for organizing, providing, and financing healthcare until 2023, when the organizing responsibilities were centralized to the regional level. Within the Norwegian system, the State owns the five regional health authorities, which in turn owns the hospitals, while general practice services, including GPs, are based at the municipality level. The Icelandic health care system is divided into two with a strong emphasis on national level. The Ministry of Health is responsible for financing, setting the framework and delivery of all types of health care. In Sweden, the main responsibility for healthcare financing and organization lies in 21 independent regions. In addition, there are constitutional limitations at the national level on minister rule in Sweden, with greater independence for public authorities, that precluded the government from enforcing similarly swift actions taken by governments in many other countries.^32^ In all Nordic countries except Iceland, local or regional authorities have quite far-reaching responsibilities. The role of primary care varies between countries, with occupational healthcare playing a larger role in providing primary care services for the employed population in Finland.^14^

*Supplemental Table S2: Definitions of COVID-19 waves*

| Wave | Dates |
| --- | --- |
| 1 | March- April 2020 |
| 2 | September 2020- May 2021 |
| 3 | July 2021- October 2021 |
| 4 | November 2021- February 2022 |

| *Supplementary Table S3: Investigated policies and their scales. Adopted from OxGCRT.^33^* | |
| --- | --- |
| **Policy** | **Nominal scales** |
| School closing  (Closing of schools and/or higher education) | 1. No measure 2. Recommended closing 3. Required closing at some level 4. Required closing all levels |
| Workplace closing-  Closing of workplaces | 1. No measure 2. Recommend closing or recommend remote working 3. Required closing or remote working for some sectors or categories of workers 4. Require remote working for all non-essential workplaces (i.e., hospitals grocery stores etc.) |
| Cancel public events-  Records cancellation of public events | 1. No measure 2. Recommend cancelling 3. Require cancelling |
| Restrictions on gatherings-  Record limits on gatherings | 1. No measure 2. Restrictions on very large gatherings (>1000 people) 3. Restrictions on gatherings between 101-1000 people 4. Restrictions on gatherings between 11-100 people 5. Restrictions on gatherings of ≤10 |
| Close public transport-  Record closing of public transport | 1. No measure 2. Recommend closing or reduce volume/route/means of transport 3. Require closing or prohibit most citizens from use |
| Stay at home requirements-  Record orders to “shelter-in-place” or otherwise confine to the home | 1. No measure 2. Recommend not leaving home 3. Require not leaving home with exceptions for daily exercise, grocery shopping and essential trips 4. Require not leaving house with minimal exceptions |
| Restrictions on internal movement-  Records restrictions on internal movement between cities/regions | 1. No measure 2. Recommend not to travel between regions/cities 3. Internal movement restricted |
| International travel-  Records restrictions on international travelers traveling to the country not necessarily for the countries’ own citizens | 1. No measure 2. Screening arrivals 3. Quarantine arrivals from some or all regions 4. Ban arrivals from certain regions. 5. Ban all regions/ total border closure |
| Public information campaigns-  Record presence of public information campaigns | 1. No campaign 2. Public officials urging caution 3. Coordinated public information campaigns |
| Testing policy-  Record policy on who has access to testing for ongoing infection PCR-tests not immunity tests | 1. No testing policy 2. Only those who have symptoms and meet specific criteria s.a. key workers, inpatients, part of contact tracing. 3. Testing of anyone with symptoms 4. Open public testing |
| Contact tracing-  Record policy on contact tracing after a positive diagnosis | 1. No contact tracing. 2. Limited contact tracing not done for all infections. 3. Comprehensive contact tracing done for all identified infections |
| Facial coverings-  Record policy on the use of face masks outside the home | 1. No policy 2. Recommended 3. Required in some specified public places when physical distancing is not possible 4. Required in all public places when physical distancing is not possible 5. Required outside the home at all times regardless of location or presence of people |
| Vaccine policy-  Record policies for vaccine delivery to different groups | 1. No vaccine available 2. Available to one of the following groups: key workers/ clinically vulnerable groups/ elderly 3. Available to two of the aforementioned groups 4. Available to all aforementioned groups 5. Available to all groups plus partially available to select broad groups and ages 6. Universal availability |


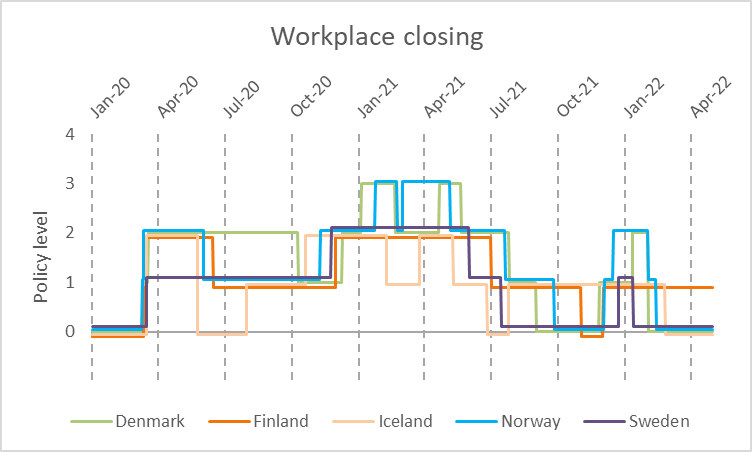


Figure S1: Workplace closing policy. 0-No measure, 1-Recommend closing or recommend remote working, 2- Required closing or remote working for some sectors or categories of workers, 3-Require remote working for all non-essential workplaces (i.e., hospitals grocery stores etc.)


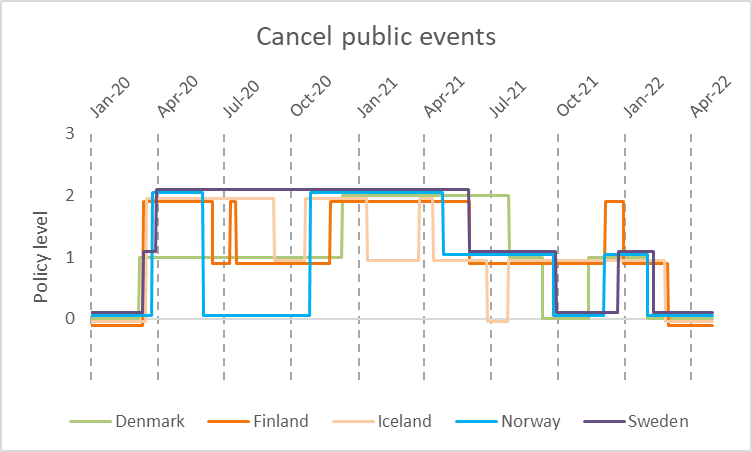


Figure S2: Public event restriction policy. 0-No measure, 1-Recommend cancelling, 2-Require cancelling


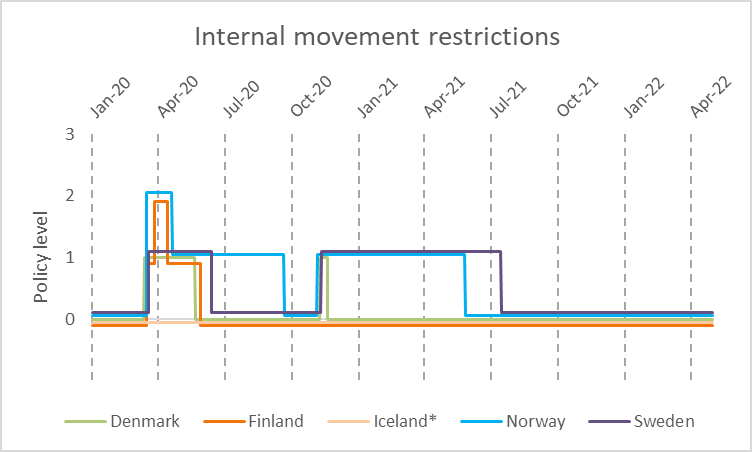


Figure S3: Internal movement retractions. 0- No measure, 1-Recommend not to travel between regions/cities. 2-Internal movement restricted


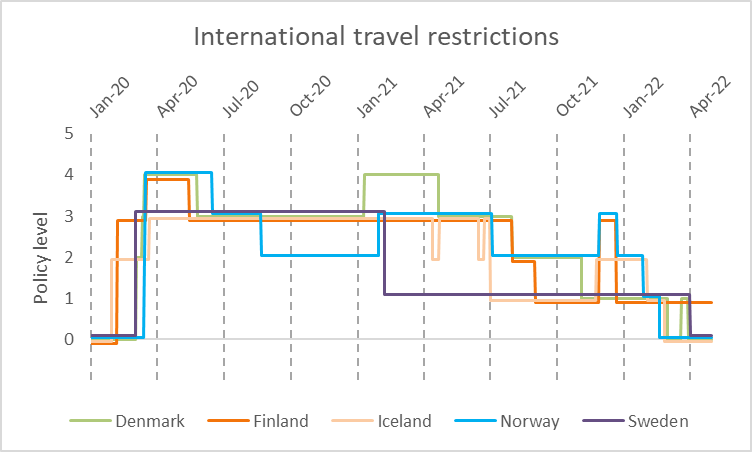


Figure S4: International travel restrictions 0-No measure, 1-Screening arrivals, 2-Quarantine arrivals from some or all regions, 3-Ban arrivals from certain regions, 4-Ban all regions/ total border closure.


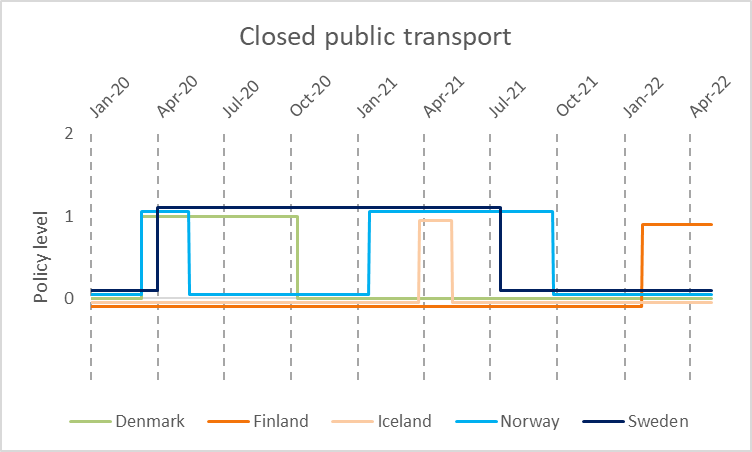


Figure S5: Changes in closure of public transportation policy. 0-No measure; 1-Recommend closing or reduce volume/route/means of transport; 2-Require closing or prohibit most citizens from use

Figures S6-S15 illustrate the government response index plotted against two epidemiological outcomes.

Figure S6: Sweden plot, the daily change in average government response index and hospital admissions.

S7: Norway plot, the daily change in average government response index and hospital admissions

S8: Denmark plot, the daily change in average government response index and hospital admissions

S9: Iceland plot, the daily change in average government response index and hospital admissions

S10: Finland plot, the daily change in average government response index and hospital admissions

S11: Sweden plot, the daily change in average government response index and deaths

S12: Norway plot, the daily change in average government response index and deaths

S13: Denmark plot, the daily change in average government response index and deaths

S14: Finland plot, the daily change in average government response index and deaths

S15: Iceland plot, the daily change in average government response index and deaths
